# Supplementary material for: Evaluating the Perceived Health-Related Effectiveness of ‘The Daily Mile’ Initiative in Irish Primary Schools
Source: Healthcare (Basel). 2024 Jun 27;12(13):1284. doi: 10.3390/healthcare12131284 (PMC11240888; doi:10.3390/healthcare12131284)
Supplement: Supplementary file 1 [file healthcare-12-01284-s001.zip › File S1_Questionnaire information.pdf]

File S1. Questionnaire information

Section	Synopsis	RE-AIM components	Question type (n)
Section A	Percentage of Irish primary schools registered as TDM participants.	Reach	N/A*
Section B	Examined perceptions associated with the introduction, delivery and long-term uptake of TDM.	Adoption, implementation and maintenance.	Open-ended (3), multiple choice (9) & 5-point Likert-scale (4)
Section C	Participants' rated their level of agreement with TDM's potential impact on a list of health-related outcomes (n = 9) and were also encouraged to volunteer any additional benefits not displayed.	Effectiveness	5-point Likert-scale (1) and open-ended (1)
Section D	Participants rated their level of agreement with barriers (n = 9) that may restrict implementation and were also invited to volunteer any additional barriers not displayed.	Implementation and maintenance	5-point Likert-scale (1) and open-ended (1)
Section E	Participants were invited to volunteer any information relating to TDM that they perceived as important.	Reach, effectiveness, adoption, implementation & maintenance	Open-ended (1)

*Reach was examined by calculating the number of schools registered as TDM participants [11], relative to the number of primary schools in Ireland [44].

The Daily Mile Foundation. Starting School. Available online: https://thedailymile.ie/ (accessed on 5 November 2022).

School Days. Find Primary Schools in Ireland by County. Available online: https://www.schooldays.ie/articles/primary-schoolsin-ireland-by-county. (accessed on 10 August 2022).
