# Supplementary material for: Evaluating the Perceived Health-Related Effectiveness of ‘The Daily Mile’ Initiative in Irish Primary Schools
Source: Healthcare (Basel). 2024 Jun 27;12(13):1284. doi: 10.3390/healthcare12131284 (PMC11240888; doi:10.3390/healthcare12131284)
Supplement: Supplementary file 1 [file healthcare-12-01284-s001.zip › File S2_Questions from interviews and focus groups.pdf]

File S2. Questions from interviews and focus groups

No.	Principal Interview	Teacher Focus Group	Child Focus Group
Q1	Can you describe how the implementation of TDM has gone since it was first introduced in your school?	Can you describe how the implementation of TDM has gone with your class since it was first introduced in your school?	Can you please explain to me about what is happening in the picture you drew about TDM?
Q2	For you, what barriers would you associate with the successful implementation of TDM in your school?	For you, what barriers would you associate with the successful implementation of TDM with your class group?	What do you enjoy most about participating in TDM?
Q3	What adaptations or additions would you make to TDM to improve the implementation success of the initiative within your school?	What adaptations or additions would you make to TDM to improve the implementation success of the initiative with your class group?	What do you not like about participating in TDM?
Q4	How is TDM successfully implemented by various classes on a typical school day?	Please explain, how you and other teachers in your school successfully implement TDM with your class groups on a typical school day?	Is there any reason why your class would not participate in TDM on a school day?
Q5	What impact, if any, has COVID-19 had on the implementation of TDM in your school?	What impact has COVID-19 had on the implementation of TDM with your class group?	Would you like your class and school to continue participating in TDM?
Q6	Would you like to see your school commit to implementing TDM in the long-term?	Would you like to continue implementing TDM with your class in the long-term?	N/A
