# Supplementary material for: Evaluating the Perceived Health-Related Effectiveness of ‘The Daily Mile’ Initiative in Irish Primary Schools
Source: Healthcare (Basel). 2024 Jun 27;12(13):1284. doi: 10.3390/healthcare12131284 (PMC11240888; doi:10.3390/healthcare12131284)
Supplement: Supplementary file 1 [file healthcare-12-01284-s001.zip › Table S1_School sex classification of questionnaire participants.pdf]

Table S1. School sex classification of questionnaire participants.

Gender	N	%
All Boys	10	5.2
All Girls	14	7.3
Co-Educational	167	87.4
